# Articular Cartilage—From Basic Science Structural Imaging to Non-Invasive Clinical Quantitative Molecular Functional Information for AI Classification and Prediction

**DOI:** 10.3390/ijms241914974

**Published:** 2023-10-07

**Authors:** Bodo Kurz, Thomas Lange, Marita Voelker, Melanie L. Hart, Bernd Rolauffs

**Affiliations:** 1 Department of Anatomy, Christian-Albrechts-University, Otto-Hahn-Platz 8, 24118 Kiel, Germany; 2 Medical Physics Department of Radiology, Faculty of Medicine, Medical Center—Albert-Ludwigs-University of Freiburg, 79085 Freiburg im Breisgau, Germany; thomas.lange@uniklinik-freiburg.de; 3 G.E.R.N. Research Center for Tissue Replacement, Regeneration & Neogenesis, Department of Orthopedics and Trauma Surgery, Faculty of Medicine, Medical Center—Albert-Ludwigs-University of Freiburg, 79085 Freiburg im Breisgau, Germany; marita.voelker@uniklinik-freiburg.de (M.V.); melanie.lynn.hart@uniklinik-freiburg.de (M.L.H.)

**Keywords:** cartilage, articular cartilage, imaging, structural targets, quantitative MRI, Raman, SHG, p-SHG, CARS, TPEF, OCT, PS-OCT, AFL, AI, optical biopsy, superficial chondrocyte spatial organization (SCSO)

## Abstract

This review presents the changes that the imaging of articular cartilage has undergone throughout the last decades. It highlights that the expectation is no longer to image the structure and associated functions of articular cartilage but, instead, to devise methods for generating non-invasive, function-depicting images with quantitative information that is useful for detecting the early, pre-clinical stage of diseases such as primary or post-traumatic osteoarthritis (OA/PTOA). In this context, this review summarizes (a) the structure and function of articular cartilage as a molecular imaging target, (b) quantitative MRI for non-invasive assessment of articular cartilage composition, microstructure, and function with the current state of medical diagnostic imaging, (c), non-destructive imaging methods, (c) non-destructive quantitative articular cartilage live-imaging methods, (d) artificial intelligence (AI) classification of degeneration and prediction of OA progression, and (e) our contribution to this field, which is an AI-supported, non-destructive quantitative optical biopsy for early disease detection that operates on a digital tissue architectural fingerprint. Collectively, this review shows that articular cartilage imaging has undergone profound changes in the purpose and expectations for which cartilage imaging is used; the image is becoming an AI-usable biomarker with non-invasive quantitative functional information. This may aid in the development of translational diagnostic applications and preventive or early therapeutic interventions that are yet beyond our reach.

## 1. Introduction

Articular cartilage imaging is highly relevant in both basic science and clinical medicine. In basic science, imaging aims to better understand structure–function relationships in cartilage development, maintenance, degeneration, and injury, and the monitoring of diseases, interventions, potential drugs, and drug carriers. Clinical medicine, on the other hand, has more practically oriented aims, using articular cartilage imaging to diagnose human disease for preoperative planning and postoperative monitoring and as an essential element of clinical trials and artificial intelligence (AI) input, with a focus on the evolving role of cartilage imaging in early disease detection.

Over the last few decades, the imaging of articular cartilage has undergone profound changes in which novel technologies and significant improvements in the existing imaging technologies have led to exciting new possibilities but also to important changes in the purpose of imaging and the associated expectations. Medical imaging, which focuses on clinical analysis and medical intervention, encompasses X-ray radiography, magnetic resonance imaging (MRI), ultrasound (US), endoscopy, and nuclear medicine techniques such as positron emission tomography (PET) and single-photon emission computed tomography (SPECT); these techniques have all been used to image articular cartilage clinically. In basic science, biological imaging is largely based on light, fluorescence, and confocal laser microscopy techniques but also on optical coherence (OCT), near-infrared imaging, fluorescence lifetime, super-resolution microscopy, and even spatially resolved proteomics and transcriptomics [1], among others. Many, but not all, of these techniques have been evaluated for imaging articular cartilage. Together, these studies have used an impressive range of technologies for articular cartilage imaging in both a medical and scientific context, which illustrates that this is an active, ever-expanding field of interest.

However, an equally impressive change can be noted in the purpose for which cartilage imaging has been and is being conducted. Whereas initially, questions pertaining to the structure of cells and tissues (structural imaging) were at the center of investigation, today’s questions pertain to structure–function relationships and utilize molecular, functional, and live imaging with the goal of visualizing chondrocytes and articular cartilage in the context of their biochemical and biomechanical functions and in health vs. early degeneration. Thus, the expectation is no longer solely to better understand the structure and associated functions of articular cartilage via imaging but, instead, to generate non-invasive, function-depicting images with quantitative information that detects the early, pre-clinical state of cartilage diseases such as primary and post-traumatic osteoarthritis (OA/PTOA [2]) and to enable preventive or early therapeutic interventions [3] that are yet beyond the horizon. In short, the aim is to use articular cartilage images as non-invasive quantitative functional information. In this context, this review summarizes (a) the structure and function of articular cartilage to highlight relevant imaging targets, (b) quantitative MRI for non-invasive assessment of articular cartilage composition, microstructure, and function with the current state of medical diagnostic imaging, (c) non-destructive imaging methods, (d) non-destructive quantitative articular cartilage live-imaging methods, (e) artificial intelligence (AI) classification of degeneration and prediction of OA progression, and (f) our contribution to this field, which is the development of a future-oriented, AI-supported, non-destructive quantitative optical biopsy for early disease detection [4] that operates on a digital tissue architectural fingerprint.

## 2. What Are the Targets for Imaging of Articular Cartilage?

Articular cartilage is a viscoelastic tissue that provides a smooth and lubricated surface for joint movement, which also plays a key role in the absorption and dissipation of loads to the underlying subchondral bone [5]. Here, we highlight some of the major components and features of articular cartilage, which are the primary targets for imaging, especially with respect to the diagnosis of early and late changes in tissue function and structure due to joint degeneration or injury (Table 1).

**Figure 1:**
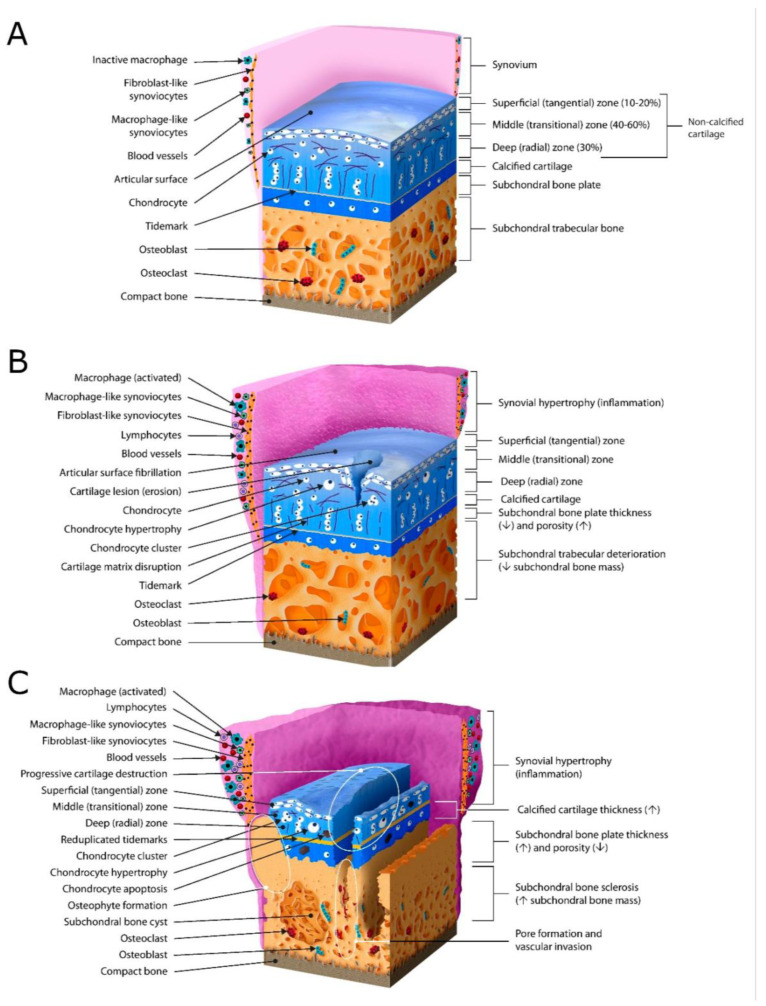
Microarchitectural and histologic changes in articular cartilage, subchondral bone, and synovium in OA. (**A**) Representation of normal joint structure and normal histologic state of the main tissue involved in OA; (**B**) early–stage OA changes in articular cartilage, subchondral bone, and synovium. Shallow cartilage erosions of the superficial tangential zone and middle transitional zone causing disruption of the ECM can be seen. Secondary chondrocyte hypertrophy and clustering is present as well. Changes to other joint tissues, mainly the reduction in subchondral bone mass, synovial thickening, and inflammatory cell (lymphocyte) infiltration, together with changes in cellular activity and count, are depicted. (**C**) Late–stage OA changes in involved tissues. Full–thickness cartilage erosions reaching the subchondral bone, chondrocyte apoptosis, subchondral bone sclerosis, and osteophyte and subchondral cyst formation alongside vascular infiltration are shown. Further synovial thickening with immune cell infiltration and increased vascularization is also seen. This figure is a reprint of Figure 2 that was originally published in [6]. No special permission is required to reuse all or part of the article published by MDPI, including figures and tables. For articles published under an open access Creative Common CC BY license, any part of the article may be reused without permission.

Figure 1 gives an overview of the microarchitectural and histological changes in articular cartilage, subchondral bone, and synovium that occur in OA. The authors would like to point out that a few OA-relevant features are not included in Figure 1, e.g., relevant anatomical characteristics such as the negatively charged glycosaminoglycans and OA-related changes in the joint surface-specific superficial chondrocyte spatial organization (SCSO), cartilage swelling that occurs due to collagen network damage in OARSI grade 1, cell death due to necrosis and/or apoptosis that occurs due to trauma and/or OA, and, finally, subchondral edema and/or cysts, which will be discussed as possible clinical imaging targets in the sections below.

### 2.1. Current Commonly Used Histological Reference for Articular Cartilage Imaging

Whenever new imaging techniques are being introduced in the clinical setting, they must be standardized and correlated to the existing understanding of the disease and methods being used. One of the main ways of grading pathological changes in articular cartilage, next to arthroscopic evaluation, is standardized histomorphologic assessment of tissue biopsies that are currently being used in animal models, in vitro tissue studies, or whenever samples can be harvested from patient joints [2,7]. One of the most commonly used grading systems is the OARSI histopathology assessment system [8], which reliably and reproducibly [9] assesses lesion depth and extent over the joint surface. Like other comparable histological grading systems, it helps to structure the temporospatial changes of the disease by splitting the sequence of alterations into grades, beginning with an intact surface and normal cartilage and cell morphology in grade 0. In grade 1, the superficial zone is still intact, but the matrix shows edema and/or superficial microscopic cracks (fibrillation), focal superficial matrix condensation, and some cell alteration (death, clusters, hypertrophy). This is followed by grade 2, showing matrix discontinuity at the superficial zone (deep fibrillation, loss of small portions of superficial matrix parallel to the surface), loss of staining for negative charges (GAG) in the upper third of cartilage, focal perichondronal increased staining in the mid-zone, and again, cell alterations. Grade 3 is characterized by vertical fissures in the mid-zone, branched fissures, loss of staining for negative charges (GAG) in the lower two-thirds (deep zone) of cartilage, new collagen formation, and again, cell alterations (especially adjacent to fissures). Grade 4 is defined by erosion and excavation with matrix/tissue loss, delamination of the superficial layer, and mid-layer cyst formation, while grade 5 cartilage histologically shows sclerotic and thicker subchondral plate bone or reparative tissue, including fibrocartilage within denuded areas (complete loss of cartilage) and microfractures with repair limited to bone surface. Grade 6 cartilage is defined by bone remodeling, including microfractures with fibrocartilaginous and osseous repair extending above the previous surface.

### 2.2. The Importance of the Extracellular Matrix (ECM) in Function and as an Imaging Target

Articular cartilage is a specialized form of hyaline cartilage that is not covered by a perichondrium. The resident cells, the articular chondrocytes, produce and maintain the ECM. Each articular chondrocyte fills up a space called lacuna, which is surrounded by a highly specialized ECM called the pericellular matrix (PCM) that is made up of specialized molecules such as collagen VI, fibromodulin perlecan, and matrilin [10]. Further away from each cell, the ECM composition changes and forms the territorial matrix first and then the interterritorial matrix [11]. Only intact cell–matrix interactions and an intact ECM provide conditions for maintenance of the tissue. Therefore, the mechanical environment of chondrocytes is thought to be a crucial factor in regulating joint health, as physiological mechanical loads on cartilage cause cell deformations that are detected by mechanoreceptors on the cell surface. Through this process of mechanotransduction, mechanical signals modulate the biochemical activity of chondrocytes, inducing the biosynthesis of molecules to preserve the integrity of the tissue [6,12].

The ECM also includes proteoglycans (such as aggrecan), glycosaminoglycans (GAGs), and hyaluronan (HA). They carry high amounts of negative charges, which attract water (representing more than 70% of the tissue volume). The interaction of the collagen network and the water-binding ECM gives articular cartilage its biomechanical property. The negative charges, together with the attracted water, create a swelling pressure in the tissue, whereas the collagen network counteracts that swelling and ensures the correct density of molecules and permeability of the ECM. Proteoglycans mainly control the equilibrium stiffness of the tissue [13], but it is important to consider both (viscoelastic) collagen fibers as well as swelling pressure in the mechanical behavior of cartilage [14]. Under prolonged loading, fluid flows through and out of the tissue, and the interstitial fluid is forced to flow through the pores of the ECM, which results in friction and an increase in the hydrostatic/hydraulic pressure, depending on the creep and relaxation behavior [14]. Further compression reduces pore sizes in the ECM even more and results in even higher friction and hydrostatic pressure until the latter is high enough to stop the process of compression [12,15]. Initially, upon loading, the hydraulic pressure seems to carry most of the load in all three non-calcified zones of the tissue, whereas the osmotic swelling pressure carries most of the equilibrium load [14]. In the surface zone, where the collagen fibers are loaded in tension, the collagen network carries up to 20% of the load, which becomes increasingly important with higher strain rates of compression. Slow (or low strain rate) compressions allow the interstitial fluid to pass through the ECM, and the tissue loses some height. The constant increase in hydrostatic pressure is a basis for the buffering of the mechanical load in order to protect the stiff subchondral bone. With higher strain rates, the fluid might not be able to overcome the friction that builds up the hydrostatic pressure even faster since the fluid has to stay where it is; this phenomenon causes dynamic stiffening [16]. In such circumstances, in this faster compression, the extra loading is predominantly transported to the fibrillar matrix via rising fluid pressure, with little increase in stress in the nonfibrillar matrix, causing the fibrillar matrix to absorb the loading increment by self-stiffening. Hence, the quicker the loading, the faster the fibril stiffening until the upper elastic loading limit is reached. Therefore, very high-impact compression might pass through the cartilage unbuffered, causing the impact to fully hit the subchondral bone, thereby creating (micro-) fractures in the bone or rupturing of the collagenous network in the cartilage.

Due to the events described above, articular cartilage volume is not preserved during tissue deformation. However, the tissue returns to its original shape after removing the external loads due to the elasticity of the solid phase of the ECM and the negatively charged ECM components, which cause both an electrostatic repulsive force and the swelling pressure built within the tissue due to osmotic pressure [12]. Together, this emphasizes that the integrity of collagen and the ground substance of the ECM, as well as the water content, could be major features in cartilage imaging.

### 2.3. The Importance of the Zonal Structure in Function and as Imaging Target in Healthy and Injured Articular Cartilage

Healthy articular cartilage can structurally be subdivided into four distinct zones: the superficial zone, middle zone, deep zone, and calcified zone. These zones exhibit particular arrangements and organizations of articular chondrocytes and ECM components, mainly collagen type II (Col II) and proteoglycans. These determine together the tensile strength, flexibility, and load-bearing ability of cartilage to resist a broad range of static and dynamic forces, e.g., shear, compression, and tension (see reviews [5,12,15]). Well reviewed by Davis et al. [5], the superficial (tangential) zone represents the upper 10–20% of the tissue thickness and contains flattened articular chondrocytes and thin collagen fibrils (mainly collagen types II and IX) tightly packed and aligned parallel to the articular surface to protect deeper layers from shear stress and carry some of the load. Thus, the superficial zone serves to protect deeper zones through microscale strain field distribution and strain concentration [17], making the superficial zone or, so far, its collagen fibers a potential and functionally relevant target of cartilage imaging [18]. This zone additionally acts as a barrier regulating the diffusion transport of nutrients to the underlying cartilage structures and the ingress and egress of large biomolecules.

The middle zone represents 40–60% of the articular cartilage thickness and is characterized by sparsely distributed rounded chondrocytes, obliquely distributed thicker collagen type II fibrils, and a richer proteoglycan content. The deep (radial) zone is characterized by the lowest water concentration, with thick collagen fibrils that run perpendicular to the articular surface, causing cartilage to be arranged in columns parallel to collagen fibril orientation. Due to the high content of negatively charged proteoglycans, the deep zone is responsible for providing the greatest compressive resistance to articular cartilage.

A study by McLeod et al. [19] showed depth-dependent mechanical inhomogeneity of the elastic moduli of the ECM across the cartilage zones, yet zonal uniformity of the PCM elastic moduli in comparison. Our own studies demonstrated that structural, compositional, and functional zonal differences can cause, even in immature and, thus, relatively homogeneous cartilage, a depth-dependent response to mechanical injury [20,21]. In injured superficial zone disks, surface disruption, tissue compaction, and immediate biomechanical impairment, such as a decrease in dynamic stiffness and equilibrium moduli below the level of detection, were noted, whereas injured deeper-zone disks showed collagen crimping but remained biomechanically intact, revealing a vulnerability of the soft superficial zone to compressive injury [21]. Interestingly, the biomechanical stress that occurs during injury predetermined the structural and functional damage during cartilage fracture [20]. These and other studies demonstrate that the zonal structure of articular cartilage and the associated functions represent interesting imaging targets. For example, they have been investigated so far by a combination of microscopic MRI, polarized light microscopy, and Fourier-transform infrared imaging [22], and also by fluorescence lifetime imaging [23] and 3D second harmonic generation imaging microscopy [24].

### 2.4. The Importance of the Structure and Function as Imaging Targets in OA/Early OA

In OA, as a result of various factors (pathological loading, obesity, aging, joint instability, repetitive stress injury, inflammation, and genetic background), cartilage becomes fibrillated in the superficial zone and loses proteoglycans, causing chondrocytes to restore lost matrix components by increasing their synthetic activity [6,11]. Articular chondrocytes experience a proliferative response and gather themselves in clusters, probably to further increase their synthetic activity [25,26], but they also adjust by undergoing hypertrophic differentiation associated with increased collagen type X expression [27]. Early surface changes seen as roughening and fibrillations extend further distally, forming deep fissures and leading to cartilage delamination uncovering the calcified cartilage and the subchondral bone [6,28]. Cartilage degeneration and lesions are problematic because hyaline articular cartilage has a limited ability to regenerate in response to damage, and fibrocartilage filling may occur with a different arrangement of cells and ECM, different phenotypes of cells, and altered composition of ECM components, resulting in insufficient biomechanical properties of the repair tissue [29]. (Focal) cartilage lesions are usually addressed arthroscopically, and there are different scoring systems for grading the amount of destruction that exists, e.g., the Outerbridge Classification and the International Cartilage Repair Society Classification. Both rely on arthroscopic inspection and focus on tissue softening to dynamic probing or on visible damage to the tissue surface with blistering, fissures, partial thickness up to full thickness fissures and lesions, and complete cartilage loss with exposed or even damaged subchondral bone, or there might be defects in the subchondral bone, too [29]. Non-invasive (functional) imaging might be able to address these parameters better and quantitatively, as assessments of arthroscopic measurements have generally poor accuracy and reliability, even between experienced arthroscopic specialists [30].

Some of the pathomechanisms of mechanical injury have already been uncovered by the use of a broad range of in vitro models, which demonstrate that mechanical injury induces many OA-related tissue alterations [7], e.g., initial tissue swelling and decrease in both the compressive and shear stiffness of articular cartilage, probably due to disruption of the collagen network [31]. The tissue content of proteoglycans decreases with time due to loss of PG and an overall reduction in new ECM synthesis because of cell death or changes in the AC phenotype and ECM interaction.

Ebrahimi et al. [15] studied the structure–function relationships in human tibial cartilage at different stages of OA and used microscopic and spectroscopic measurements to characterize the zonal depth-wise structure and composition (distribution of collagen fibrils and PGs). The PG content of OA tibial cartilage was lower at the early stages of OA when compared to the healthy tissue, which progressed to deeper layers of cartilage at the later stages. The collagen content, on the other hand, showed no signs of changes in early OA but decreased at the late stage of disease in the middle and deep cartilage layers. In contrast to the collagen content, the collagen fibrils disorganize at the superficial cartilage in early OA, and this disorganization extends deeper in the tissue [15]. The authors suggest that loss of the collagen pretension in the superficial tissue during early OA is mainly due to the lower tissue swelling (caused by the loss of PGs) rather than collagen disorganization. However, the loss of collagen fibril pretension in advanced OA is suggested to be regulated by both collagen disorganization and lower tissue swelling. Extending the findings of [15], a recent study by our team demonstrated that the collagen network changed in early OA by collagen fiber thinning and the formation of fibrocartilage-like tissue and that both alterations already occurred in the macroscopically intact regions of human knee cartilage and were likely connected to processes that result in a weakened ECM [32]. Thus, zonal collagen network imaging could be a valuable tool in early OA detection.

Since cartilage matrix is impermeable to anionic molecules, owing to the negative potential of proteoglycans, anionic contrast agent-enhanced CT could help to diagnose early OA since anionic medium uptake is accelerated at sites of cartilage injury only. Cationic contrast agents also have potential in the diagnosis of cartilage damage based on the electrostatic attraction effect; using nonionic agents, the collagen concentration can be mapped on contrast-enhanced CT [33]. With increasing knowledge of the tissue composition and function of individual molecules, it might be important to visualize even specific molecule epitopes. For example, Bhatti et al. [34] used near-infrared optical imaging as a method for detecting joint damage by using targeted nanosomes (nano-sized liposomes) that carry monoclonal antibodies that can detect exposed type II collagen. These nanosomes can deliver both a detection probe and a therapeutic drug to the damaged site, which would combine the diagnosis of an ECM defect with a treatment at the same time.

### 2.5. Imaging Cartilage Thickness

Cartilage thickness is one of the most widely evaluated parameters because it best expresses the disease classically evoked in OA by cartilage erosion and is probably the easiest parameter to quantify using an automated computerized image analysis [35]. Impaired joint loading significantly affects articular cartilage ECM composition, and, as a consequence, cartilage becomes thinner with a reduced ability to absorb loads and shocks, resulting in excessive load transmission to the underlying subchondral bone [5]. Measurement of joint space width (JSW) has, for a long time, served as a surrogate measure of cartilage thickness since the latter cannot be directly visualized radiographically, whereas joint space narrowing (JSN) is the primary indicator of structural OA progression, and complete loss of JSW is an indication for joint replacement surgery [36].

However, cartilage thickness can vary, even in healthy joints. Using MR imaging and advanced digital post-processing techniques, Eckstein et al. [37] presented normative values on cartilage volume, thickness, and joint surface areas in human joints. They found relatively small differences in cartilage morphology between both limbs of the same person (approximately 5%) but large differences between individuals (CV% approximately 20%). Men display slightly thicker cartilage than women (approximately 10%) but significantly larger joint surface areas (approximately 25%), even when accounting for differences in body weight and height. The thickness appears to decrease slightly in the elderly, in particular in women, even in the absence of OA cartilage lesions. The authors compared cartilage volume, thickness, and joint surface area in the knee, ankle, and subtalar joint using sagittal MR images (3D FLASH WE) but found only moderate correlations in cartilage thickness of healthy subjects between knee and ankle [38]. Sex differences in cartilage morphology at the knee and the ankle were similar, with surface areas being −17.5% to −23.5% lower in women than in men. The authors concluded that it is, therefore, impractical to estimate knee joint cartilage loss a posteriori in cross-sectional studies by measuring the hind foot and then applying a scaling factor and that sex differences in cartilage morphology might not explain differences in OA incidence between men and women in the knee and ankle. Due to these variations, cartilage thickness might not be an ideal imaging parameter for the detection of especially early pathological changes in articular cartilage.

### 2.6. Imaging Articular Chondrocyte Volume, Shape, Phenotypes, and Spatial Arrangements

Articular cartilage is built and maintained by articular chondrocytes, which make up about 1–5% of the total tissue volume but produce all of the ECM. These cells differ in shape, function, and cellular organization, depending on the location in the tissue or the state of disease.

Classical histology has shown many times that cell shape and spatial arrangements differ in the different zones of cartilage. Flat cells are arranged parallel to the surface in the superficial (tangential) zone, spherical cells are randomly organized in the middle zone, cells are organized in columns perpendicular to the surface in the deep zone, and there are small randomly distributed cells in the calcified zone. While classical histological methods are limited in the demonstration of the heterogeneity of articular chondrocyte morphology, newer visualization of fluorescently labeled in situ methods—for example, confocal scanning laser microscopy (CLSM)—can be used to image chondrocyte heterogeneity within non-degenerate and mildly or even more degenerate cartilage [39]. Karim et al. identified different chondrocyte morphologies marked by changes in chondrocyte volume and appearance in the form of cytoplasmic processes in human femoral head cartilage, representing different phenotypes of articular chondrocytes [40].

It has long been known that articular chondrocytes lose their tissue-specific phenotype (de-differentiation) when they are isolated from the tissue and cultured in a 2D culture system, where they more or less lose their rounded shape [41]. Interestingly, it was already reported in 1981 that chondrocytes in the center of chondrocyte colonies reassume a spherical shape and that in these cells, immunofluorescence revealed type II collagen [42]. Culturing chondrocytes in a hydrogel can help them to maintain a spherical shape and retain their cellular phenotype to some extent [43], which suggests a correlation between cell morphology and function [44]. Importantly, multiple recent studies clearly demonstrated that controlling phenotypically relevant functions through controlling cell shape can be achieved by micro-patterning cell populations through geometrically defined adhesion sites. This allows for controlling morphogenesis, polarity, cellular mechanics, proliferation, migration, differentiation, stemness, cell–cell interactions, collective cell behavior, and likely immuno-modulatory properties of multiple cell types, which was reviewed in [45]. Moreover, recent evidence shows that cell morphology can be used as a biological fingerprint of phenotype in healthy, inflamed, and degenerating/diseased chondrocytes, especially when coupled to multivariate data analysis methods for identifying discriminatory features [46]. Here, morphological changes correlated with changes in the expression of ECM- and inflammatory-regulating genes. Some of these morphological changes have been linked to early articular cartilage degradation [47]. Lauer et al. [48] recently reviewed how the cytoskeleton, which has a major impact on cell morphology, regulates the phenotype and function of articular chondrocytes, making cell morphology an important target for imaging cell shape and related function.

More and more research has highlighted that articular chondrocytes can be divided into different stages of activation, maturation, or even subpopulations, which means that there are different phenotypes of cells distributed throughout the tissue. One subpopulation of articular chondrocytes is chondrogenic stem/progenitor cells (CSPC), which increase in number and activation after cartilage injury [49,50,51]. In late-stage OA, Ji et al. defined up to seven subpopulations of articular chondrocytes by single-cell RNA-seq analysis, including homeostatic, proliferative, effector, regulatory, pre-hypertrophic, hypertrophic, and fibrocartilage articular chondrocytes [52]. For the diagnosis and understanding of cartilage degeneration, it would be helpful to identify the different phenotypes of articular chondrocytes by non-invasive imaging.

Rolauffs et al. analyzed the arrangement of fluorescently labeled nuclei of superficial-zone chondrocytes in a top-down view of non-degenerated human joint cartilage (shoulder, elbow, knee, and ankle) and demonstrated that articular chondrocytes occurred in four distinct patterns of strings, clusters, pairs, or single chondrocytes and that each articular joint surface was dominated by only one of these four patterns. Moreover, specific patterns correlated with specific diarthrodial joint types [25]. Additionally, they identified a distinct spatial reorganization of human superficial chondrocytes that responded by proliferating in response to distant early (grade 2) OA lesions, suggesting that proliferation that had occurred distant from the lesions may serve to recruit metabolically active units in an attempt to repair the focal damage [53]. These disease-specific spatial patterns of cell organization indicate specific image-based phases of OA development. The spatial organization of the joint surface—ranging from single cells to cells arranged in strings, double strings, or clusters, up to a diffuse cell distribution—therefore seems to be a key factor in imaging for the understanding of tissue functioning or the diagnosis of early OA [54].

A critical decrease in ECM stiffness has been implicated in OA-related changes in chondrocyte phenotype [55], which illustrates that material stiffness changes are important aspects of the many OA pathomechanisms [56], which are related to tissue and cellular functions. One of our studies used hybrid fluorescence-Atomic Force Microscopy (AFM) imaging to explore articular surface degeneration in early OA across length scales [57]. Here, under native conditions, early OA-related features of cell organization of a millimeter-sized human articular cartilage sample were selected and analyzed by fluorescence microscopy on the micrometer scale but mapped down to nanometer precision by AFM under native conditions. This hybrid imaging revealed that local changes in the organization of fluorescently stained cells, used as a marker for early OA, correlated with a significant local reduction in the elastic modulus, local thinning of the collagen fibers, and a roughening of the articular surface. Thus, the current efforts of our team, which are described in more detail in Section 6, target clinical visualization and diagnostic quantification and modeling [4].

### 2.7. Imaging Articular Chondrocyte Viability, Hypocellularity, and Cell Death

Due to the low density of articular chondrocytes in healthy tissue and the low regenerative capacity of the cells [58], imaging of articular chondrocyte viability and types of cell death seems to be of high importance. The density of articular chondrocytes, however, varies in articular cartilage, even in healthy tissue. Using confocal and electron microscopy, Quinn et al. demonstrated in osteochondral explants of donors aged 20–40 years that knee cartilage has higher cell densities in the superficial zone than ankle cartilage [59]. In the transitional zone, higher cell densities are observed in association with convex vs. concave articular surfaces, without significant differences between knee and ankle cartilage. But highly uniform cell and matrix morphologies are evident throughout the radial zone in the knee and ankle; throughout the knee and ankle cartilage, chondron (cell plus pericellular matrix) density was remarkably constant at approximately 4.2 × 10^6^ chondrons/cm^3^. The authors concluded that tissue structural adaptations through variations in cartilage cell density with changing joint and biomechanical environments are performed primarily by the superficial and transitional zones, which might help to identify site-specific pathological changes or develop site-specific cartilage tissue engineering.

In the context of diagnosis, early OA is characterized by three key events, namely, a rarely considered early phase of proliferation of cartilage-resident cells, in contrast to well-established increased synthesis and degradation of ECM components and inflammation, which are associated with OA progression [60]. Thus, imaging articular chondrocyte cellularity could aid in detecting early OA processes that are currently beyond clinical detection limits.

In the context of PTOA, David et al. examined disease progression in a murine model of medial meniscus destabilization (DMM) and observed a drastic loss in chondrocyte number (by day 3 after DMM), surface damage (at 7 days), and cartilage erosion (at 84 days) in the affected region of the medial tibial plateau [61]. The authors suggest that joint instability and injury may trigger immediate (<3 days) processes within a population of chondrocytes that direct the initiation and progression of PTOA. Therefore, the development of detection methods for live imaging of cell viability in humans seems to be needed.

There are different types of cell death in articular cartilage that have recently been reviewed by Riegger and Brenner for post-traumatic cartilage and OA [50]. These include autophagic cell death, apoptosis, and varying forms of necrosis and might lead to tissue hypocellularity, a feature seen in OA. The authors state that in the context of cartilage injury, both progression and modus of trauma-associated cell death can be assigned to the time after impact as well as the location of the cells, implying a certain degree of spatiotemporality. The mechanical impact results either in immediate cell death, namely necrosis, which is characterized by sudden plasma membrane disruption and subsequent inflammatory and other various pathogenetic responses, or apoptosis, which does not lead to uncontrolled release of molecules and is, therefore, considered a non-inflammatory mode of regulated cell death. The authors also previously showed that necroptosis, which occurs as a regulated form of necrosis, occurs in highly degenerated human cartilage, implying a potential role of necroptosis in OA disease. This was in conjunction with one of our own studies that investigated cellularity as a function of the joint surface Collins grade and OA lesion severity, which revealed that human condylar cellularity decreased in Collins grade 3 in severe OA lesions to values below 50%. Rat condylar and talus cellularities followed the same trend and showed greater decreases in cellularity [62]. The human and rat cellularity decreases occurred in conjunction with changes in spatial arrangements (discussed in Section 6).

Imaging the amount and distribution of cellularity, autophagy, and apoptosis in diagnosis and the course of treatment seems to be an important aspect since it has been shown in many ways that mechanically induced apoptosis in articular chondrocytes can be prevented by inhibition of apoptotic pathways by anti-oxidative treatment, caspase inhibitors, or other ways [63]. Autophagy plays a protective role on cells under abnormal physiological conditions, and, at present, it is generally believed that autophagy as an adaptive response can reduce cell death in the early stage of OA. However, with the development of OA, excessive autophagy may also cause cell death, which is why autophagy as a therapeutic target for OA shows broad clinical prospects [64] and would be a promising imaging target.

In general, chondrocyte death leads to hypocellularity [31,65], which not only comes along with reduced capacities for ECM production but also promotes cell clustering that (a) is commonly regarded as a possible compensatory response of the cells, even though the cells repair tissue [50,66], and (b) could be used quantitatively as an imaging target [4,67,68]. Such repair tissue is not articular cartilage but rather fibrocartilage [69], which has inferior biomechanical and biochemical properties [69,70,71] and poorer wear characteristics [72]. In their injury animal model, Riegger and Brenner found hypocellularity and cell cluster formation mainly located in the superficial zone in close proximity to the mechanical impact [51]. Apoptosis clearly occurs in OA cartilage, but the relative contribution of chondrocyte apoptosis in the pathogenesis of OA is difficult to evaluate. It is not clear whether chondrocyte apoptosis is the inducer of cartilage degeneration or a byproduct of cartilage destruction; chondrocyte death and matrix loss may form a vicious cycle, with the progression of one aggravating the other [73]. Nevertheless, this text section clearly demonstrates that imaging cellularity, autophagy, and apoptosis would be diagnostically valuable.

### 2.8. Imaging the Pericellular Matrix (PCM)/Chondron

While the effects of OA on the cartilage ECM have been well recognized, it is now becoming apparent that the onset of the disease may be initially reflected in the matrix region immediately surrounding the chondrocytes, termed the pericellular matrix (PCM) [10]. The PCM, along with the enclosed chondrocyte, termed the “chondron”, acts as a mechanical protection shield and as a critical transducer or “filter” of biochemical and biomechanical signals for the chondrocyte, serving to help regulate the homeostatic balance of chondrocyte metabolic activity in response to environmental signals. The PCM is rich in perlecan, aggrecan monomers and small aggregates, hyaluronan, biglycan, types VI and IX collagen, and fibronectin [74]. Youn et al. made three-dimensional reconstructions of intact, type VI collagen, fluorescently labeled chondrons from stacks of confocal images recorded in situ from the superficial, middle, and deep zones of porcine articular cartilage of the medial femoral condyle (type VI collagen is a marker molecule of the PCM) [75]. Significant variations in the shape, size, and orientation of chondrocytes and chondrons were observed within different cartilage depths, revealing flattened discoidal chondrons in the superficial zone, rounded chondrons in the middle zone, and elongated, multicellular chondrons in the deep zone. The shape and orientation of the chondrons appeared to reflect the local collagen architecture of the interterritorial matrix, which varies significantly with depth. Rothdiener et al. compared human OA chondrons with OA chondrocytes in a head-to-head comparison and demonstrated that the OA chondrons displayed superior biosynthesis and mRNA expression of tissue engineering and phenotype-relevant genes and a significant survival advantage in hydrogel culture [76]. Using the destabilization of the DMM murine model of PTOA, Chery et al. showed that decreases in PCM micromechanics are apparent as early as 3 days after injury [77] and that this precedes changes in the bulk ECM properties and overt indications of cartilage damage, suggesting that changes in PCM micromechanobiology are leading indicators of the initiation of PTOA. Guilak et al. found that alterations in the PCM morphology in OA cartilage correlated with the loss of mechanical properties [10]. They used images of immunofluorescence labeling of type VI collagen. Growth factor-induced articular chondrocyte proliferation and subsequent changes in the cellular arrangement of articular chondrocytes in human articular cartilage are also accompanied by loss of collagen type VI immune signaling and a micro-fibrillar collagen intensity decrease (shown by second harmonic generation microscopy) in the PCM, which also suggests that the PCM is an interesting target for imaging of early changes in cartilage tissue [67]. Therefore, novel sample processing protocols are being developed to visualize human articular cartilage chondrons using micro-computed tomography (μCT) in order to develop and validate an algorithm to semi-automatically quantify chondrons/chondrocyte 3D morphology and compare the differences in chondron morphology between intact and OA cartilage [78].

### 2.9. Calcified Zone, Tidemark, and Imaging Hypertrophy

The calcified zone (CZ) is characterized by mineralized cartilage tissue with hypertrophic chondrocytes, has a high content of collagen type X (Col X), is mostly avascular, and anchors the collagen fibrils from the deep zone of the cartilage to the subchondral bone, providing optimal integration [5]. It is more dense than bone and 10-fold thinner than the overlying hyaline articular cartilage [79]. It acts as a force transmitter and is important for reducing stress concentrations at the cartilage–bone interface [80]. The CZ is approximately 100 times stiffer than the overlying hyaline cartilage and 10 times less stiff than the underlying subchondral bone [81,82]. Alterations in the CZ thickness are associated with increased risk of joint injury [83]. Madi et al. used fast, low-dose, pink-beam synchrotron X-ray tomography combined with mechanical loading at nanometric precision for in situ imaging of hypertrophic cells and mechanical strain in intact untreated joints under physiologically realistic conditions in a spontaneous model of OA using the STR/Ort mouse [84]. The authors showed, in OA-prone joints, greater chondrocyte hypertrophy, abnormally high strains in the calcified cartilage, localized calcified cartilage cracking, and development of tissue strains consistent with a stiffer articular construct, features which have been associated with OA. The authors described larger hypertrophic chondrocyte lacunar volumes in the CZ of 20-week-old STR/Ort OA joints than in age-matched control healthy joints. After 40 weeks, OA mouse joints demonstrated a significantly greater CZ thickness. The authors concluded that greater levels of calcified cartilage chondrocyte hypertrophy predispose greater strain concentration, load-induced micro-cracking, and OA.

The layer between the deep articular cartilage and the calcified zone appears histologically as a basophilic line and is called the tidemark or hyaline-calcified cartilage interface. This is not a single line but a complex three-dimensional structure where the cartilage layer penetrates the calcified layer and eventually reaches the subchondral bone plate in OA. This allows material exchange across the tidemark or cement line (the junction between bone and the calcified zone of the cartilage) or even direct cell-to-cell communication among chondrocytes, osteocytes, and osteoclasts (see [33,85] and our section on the subchondral bone below). Reviewed by Cucchiarini et al. [11], recent experimental data suggest that hypertrophic chondrocytes within the calcified cartilage may transdifferentiate into osteoblasts, thus directly obtaining the capacity for bone formation, resulting in ossification and an accelerated advancement and duplication of the tidemark as well as increased thickness of the CZ. This process may increase the mechanical stress in the deeper zones of the hyaline articular cartilage, contributing to the acceleration of OA. The tidemark has also been found to shift slowly toward the joint space during aging [86].

Laverty et al. re-examined X-ray micro-computed tomography (µCT) image sets of the third carpal bones (C3) of standard-bred racehorses with naturally occurring repetitive loading-induced OA for the presence of high-density mineral infill (HDMI) in CZ cracks and possible high-density mineralized protrusions (HDMP) from the tidemark into the hyaline articular cartilage [87]; these are structures that are candidate causes for mechanical tissue destruction in OA at the CZ/hyaline tissue interface. The authors showed that 20 µm µCT resolution in 10 mm diameter samples was sufficient to detect HDMI and HDMP structures, which are usually lost histologically upon tissue decalcification.

During OA, healthy articular chondrocytes can become hypertrophic and undergo terminal differentiation, comparable to the processes in the growth plate [88], which is considered a crucial hallmark in OA progression [89]. The molecular events behind this have been reviewed by Neefjes et al. [90]. Healthy chondrocytes express proteins such as Indian Hedgehog (IHH) and parathyroid hormone-like protein (PTHrP) that regulate hypertrophic differentiation via a negative feedback loop. When signaling by these factors is altered, chondrocytes become hypertrophic, show an increased cell size at the early stages of hypertrophic differentiation, and switch to a new genetic program, with the expression of new proteins like collagen type X (COL10A1) and matrix metalloproteinase 13 (MMP-13), and they actively mineralize their surroundings with protein-like alkaline phosphatase (ALPL). These events and the resulting degenerative phenotype result, in part, from signaling processes that originate from or converge on the cytoskeleton, illustrating that actin dynamics regulating processes decisively control the chondrocyte phenotype [48]. In this context, changes in cell volume and mineralization by hypertrophic articular chondrocytes might be interesting parameters for imaging of cartilage degeneration.

### 2.10. Imaging Subchondral Bone

Subchondral bone can be divided into two parts anatomically: (a) the subchondral bone plate (SBP) as a compact, polyporous calcified plate crisscrossed by multiple vessels and nerve fibers and (b) subchondral bone trabeculae, which are cancellous bony structures subjacent to the SBP [33]. The osteochondral junction is located between the deep layers of the articular cartilage and the underlying subchondral bone, which comprises the non-calcified deeper articular cartilage, the tidemark, the calcified layer/zone, the cement line, and the subchondral bone where it seals off epiphyseal sensory nerves and blood vessels from the deep layer of articular cartilage [91]. Despite this seal, experiments on small molecular diffusion have revealed the existence of direct molecular signaling linking cartilage and subchondral bone [92], and this crosstalk seems to increase in OA, suggesting that the subchondral bone microenvironment is involved in cartilage degeneration in OA and vice versa [6,33]. As reviewed by Donell, early in the evolution of OA, osteoclasts penetrate the osteochondral junction, allowing new blood vessels and sensory nerves to infiltrate the deep layer of the articular cartilage [91], a mechanism that also causes pain in OA [6].

As an early sign of OA, bone marrow lesions/edema (BMLs) indicate pathological subchondral bone remodeling, which is recognized by high-signal areas on T2-weighted or proton density-weighted MRI scans due to higher water content. In approximately two-thirds of OA patients with subchondral BMLs, cartilage erosion occurs around the superficial area of the subchondral BMLs, indicating the underlying linkage between subchondral BMLs, cartilage degeneration, and OA progression [33,93]. Longitudinal studies following anterior cruciate ligament rupture suggest that subchondral bone changes may precede articular cartilage degeneration in OA initiation [94]. Subchondral BML, including angiogenesis (increased vascularization on MRI), might develop earlier than cartilage degeneration, and recent clinical data have shown a strong association between BMLs and cartilage damage in the tibial plateau, indicating the diagnostic value of predicting the degenerative status within the osteochondral unit [33,95].

At later stages of OA, subchondral bone cysts develop at sites of former bone marrow BMLs, a hallmark of advanced OA [96]. Additionally, the development of subchondral sclerosis occurs along with osteophyte formation due to persistent abnormal mechanical stresses, which create a cellular and biomolecular response to microfractures in the subchondral bone and osteochondral junction [91]. The subchondral bone plate thickness (SBPT) is an indicator of sclerosis [35], but bone volume changes at the trabecular level can also be considered as secondary indices of bone remodeling; the amount of bone increases but the mineral density is reduced, with fewer but thicker trabeculae [97]. Osteoblasts in late-stage OA secrete a type I collagen α1 homotrimer phenotypically distinct from the normal α1/α2 heterotrimer, possessing a reduced affinity for calcium, which resolves the paradox of increased subchondral bone density relative to decreased bone mineralization and stiffness in late OA [98,99]. Thus, imaging the subchondral bone at various resolutions could aid the clinical detection of earlier OA states.

## 3. Quantitative MRI for Non-Invasive Assessment of Articular Cartilage Composition, Microstructure, and Function

Due to its excellent soft tissue contrast compared to X-ray radiography, MRI has been established as a gold standard for the non-invasive evaluation of human cartilage in vivo. Morphological changes due to higher-order cartilage defects can be visualized with conventional MRI protocols and assessed with semi-quantitative scoring systems [100]. However, microscopic cartilage lesions and early-stage degeneration cannot be detected with these standard techniques [101]. For detection of these subtle changes, quantitative evaluation of cartilage composition and microstructure is required. To this end, more advanced MRI methods have been developed [102]. In particular, relaxometric measurements via T_2_ mapping, T_1ρ_ mapping, and dGEMRIC (delayed Gd-enhanced MRI of cartilage) offer great potential since these relaxation parameters can serve as objective biomarkers in clinical studies. Furthermore, technically challenging methods such as diffusion-weighted imaging, sodium MRI, and gagCEST were recently proposed to complement morphological cartilage MRI. These advanced methods are more specific with respect to certain cartilage constituents like collagen or proteoglycans, and their quantitative nature enables the objective evaluation of disease progression in longitudinal studies.

The determination of the relaxation parameters T_1_, T_2_, and T_1ρ_ requires a series of measurements with varying evolution intervals. For mapping of the longitudinal relaxation parameter T_1_, the evolution time between an inversion pulse and the excitation pulse of the MRI sequence is varied, whereas, for mapping of the transverse relaxation time T_2_, the evolution interval between excitation and signal acquisition (echo time) is incremented. T_1ρ_ mapping can be realized via irradiating spin-lock pulses of varying duration prior to excitation. Since, in any case, repeated measurements are required, relaxometric MRI protocols are typically much longer than standard morphological scans. The three above-mentioned relaxation parameters show different sensitivity with respect to the cartilage constituents collagen and proteoglycans, as well as the water bound in the cartilage matrix. The T_2_ relaxation time depends on the water content of the cartilage as well as on collagen content, orientation, and anisotropy. Elevated T_2_ values are typically indicative of cartilage degeneration. However, T_2_ is less sensitive to changes in GAG content. T_1ρ_ relaxation mainly depends on water molecules near large immobile macromolecules such as the proteoglycans and is, therefore, more sensitive to a change in GAG content than T_2_ relaxation [103]. A high GAG concentration is, therefore, associated with low T_1ρ_ values and vice versa. Due to the spin lock pulses, T_1ρ_ mapping typically entails large energy deposition in the body, which can give rise to local tissue heating. Nishioka et al. validated in vivo T_2_ and T_1ρ_ mapping in knee OA patients against histological grading of subsequently harvested osteochondral samples [104]. They demonstrated that T_2_ and T_1ρ_ values significantly correlated with the degree of cartilage degeneration as measured by the OARSI grade. However, only T_1ρ_ mapping yielded significantly different relaxation times for OARSI grades in the range between grade 1 and grade 4.

The native T_1_ contrast is less useful for the evaluation of cartilage composition and integrity than T_2_ and T_1ρ_. Ex vivo studies revealed inferior classification accuracy based on OARSI grading for T_1_ mapping compared with T_2_ mapping [105]. However, T_1_ mapping can be rendered very sensitive to the GAG content by using a gadolinium-based contrast agent. This technique is called delayed gadolinium-enhanced MRI of cartilage (dGEMRIC). The contrast agent Gd(DTPA)^2−^ is paramagnetic and, therefore, gives rise to strongly reduced T_1_ relaxation times. Since both the GAGs and the molecules of the contrast agent are negatively charged, the Gd(DTPA)^2−^ concentration is inversely proportional to the GAG concentration. Reduced GAG content due to cartilage degeneration therefore manifests itself through reduced T_1_ values. For relaxometric cartilage examinations, the contrast agent is injected intravenously. As cartilage tissue is avascularized and the contrast agent accumulates in the cartilage via diffusion, the dGEMRIC measurement is typically performed 1–2 h after contrast agent injection. Therefore, a concentration gradient between superficial and deeper cartilage layers has to be taken into account. For quantitative evaluation of the GAG content, it is recommended to perform the T_1_ mapping protocol twice—once with and once without contrast agent. In reparative cartilage, Watanabe et al. determined the difference between the relaxation rates measured before and after contrast agent injection (ΔR = ΔR_Gd_ − ΔR_native_ = 1/T_1,Gd_ − 1/T_1,native_) and demonstrated that the ratio of ∆R in cartilage repair tissue and normal hyaline cartilage correlated well with GAG concentrations measured in cartilage biopsy specimens by liquid chromatography [106]. The dGEMRIC method has been shown to have great potential for assessment of tissue healing after cartilage repair surgery, e.g., via autologous chondrocyte implantation (ACI) [107]. In contrast to T_1ρ_ mapping, which is sensitive to both GAG and collagen content, dGEMRIC measurements specifically reflect the GAG concentration. However, since contrast agent injection is an invasive and potentially risky procedure, the application of dGEMRIC should be weighed carefully.

An emerging relaxometric cartilage MRI method is T_2_* mapping based on ultra-short-TE (UTE) acquisitions [108]. The T_2_* relaxation constant reflects the combined effect of T_2_ relaxation and signal dephasing due to tissue inhomogeneity. In contrast to T_2_ mapping, UTE-based T_2_* mapping is more sensitive to changes in the calcified cartilage layer and osteochondral junction, which exhibit extremely small T_2_ and T_2_* values.

Relaxometric maps do not only contain information about cartilage composition but also about cartilage structure [109,110]. Tissue anisotropy, as observed in cartilage, comes along with more pronounced relaxation and, consequently, results in shorter relaxation times. Additionally, this effect is orientation-dependent, i.e., its strength depends on the orientation of the cartilage fibers with respect to the external magnetic field of the MRI magnet. Therefore, relaxation maps often exhibit strong regional variations in articular cartilage due to the fact that the fibers are oriented tangentially in superficial cartilage layers and radially in deep cartilage, with a transitional zone of rather isotropic tissue in between. While this anisotropic relaxation effect is typically negligible for T_1_ mapping and, consequently, dGEMRIC, it can be quite substantial for T_1ρ_ and, in particular, T_2_ or T_2_* mapping. In longitudinal relaxometric MRI studies, a consistent joint positioning within the magnet should be aimed for to avoid an orientation-dependent bias. The sensitivity of T_2_ to tissue orientation and anisotropy has been exploited to distinguish ACT repair tissue 12–15 months after surgery from normal cartilage based on the different collagen arrangements [107].

Another advanced MRI technique for the investigation of cartilage composition and microstructure is diffusion-weighted imaging (DWI) or diffusion tensor imaging (DTI), which yields the mean diffusivity and the fractional anisotropy of the cartilage as quantitative outcome measures [111]. While both GAG and collagen content contribute to the mean diffusivity, the collagen network mainly affects the fractional anisotropy. Diffusion-weighted imaging can, therefore, provide information about the quality and orientation of collagen fibers and may serve as an indicator of early-stage cartilage degeneration. Ex vivo DTI studies have demonstrated good performance in grading cartilage damage according to the OARSI score [112]. In vivo, DWI was used clinically to assess tissue quality after cartilage repair surgery in comparison with the established MOCART score [113]. However, it should be noted that technical imperfections such as subject motion and very long acquisition times can render in vivo DWI studies quite challenging.

For specifically investigating the GAG content of articular cartilage, two other advanced MRI methods were proposed: gagCEST and sodium MRI. The gagCEST technique measures the chemical exchange of protons bound in the hydroxylic residues of the GAG molecules as well as free water via spectrally selective excitation of the hydroxylic GAG resonances [114]. It can thus estimate the GAG content of articular cartilage based on the magnetization transfer asymmetry [115]. Sodium MRI is based on the detection of positively charged ^23^Na ions, which are attracted by the negatively charged GAGs and are, therefore, distributed in accordance with GAG concentration. Ex vivo studies have shown that the GAG concentration can be estimated for human studies via calculation of the fixed charge density, which is derived from ^23^Na MRI [116]. In vivo ^23^Na imaging was applied for evaluation of cartilage quality after different types of clinical cartilage repair surgery where a high correlation with the MOCART score could be demonstrated [117]. The clinical application of both gagCEST and sodium MRI, however, is encumbered by various limitations. Both techniques suffer from low sensitivity and, therefore, rely on high magnetic field strength and long scan times. Sodium MRI also requires dedicated transmit and receive coils, while gagCEST is easily impaired by magnetic field inhomogeneities and requires sophisticated data processing to robustly estimate the magnetization transfer asymmetry.

**Figure 2:**
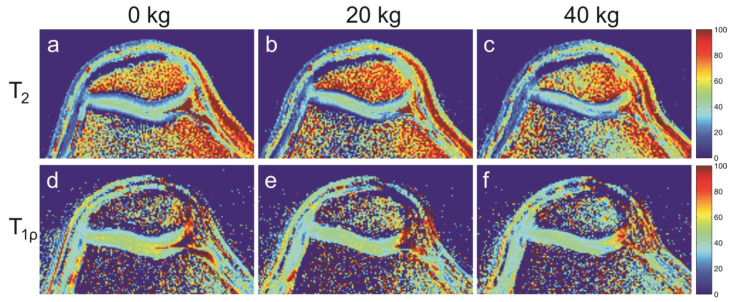
Color-coded T_2_ and T_1ρ_ maps. Shown are color-coded T_2_ (**a**–**c**) and T_1ρ_ (**d**–**f**) maps (in ms) of the patellofemoral cartilage from MRI measurements on a healthy subject performed with in situ loads of 0, 20, and 40 kg. This figure is a reprint of Figure 4, originally published in [122]. Permission for this reprint has been obtained.

Relaxometric MRI markers not only reflect cartilage composition and microstructure but also cartilage function. Nieminen et al. showed that T_2_ mapping and dGEMRIC results correlate well with the biomechanical properties (e.g., the Young’s modulus) of bovine cartilage samples [118]. In vivo knee cartilage MRI studies have also been performed with in situ loading, either with the subject in an upright position using open low-field MRI systems [119] or with high-field systems using MR-compatible loading devices [120], the latter in individuals with and without knee OA. While weight-bearing in an upright position is more physiological, high-field MRI systems with a horizontal bore enable better image resolution and can be augmented with real-time correction techniques for motion artefact suppression [121,122]. Figure 2 shows high-field T_2_ and T_1ρ_ maps of the patellofemoral cartilage acquired with different in situ loads (0/20/40 kg), which were applied via a pneumatic loading devie [122].

These results demonstrate a T_2_ and T_1ρ_ decrease in response to knee loading. Comparative T_2_ and T_1ρ_ measurements with in situ loading have been performed in healthy subjects and patients with OA, revealing a load-induced T_2_ and T_1ρ_ decrease in both cohorts [123]. Interestingly, the load-induced changes were larger in the OA group than in healthy subjects, suggesting that the cartilage matrix of OA patients may be less capable of retaining water and dissipating mechanical loading. To date, weight-bearing MRI for functional cartilage investigation has almost exclusively focused on the knee joint. However, with more advanced MRI hardware technology, functional cartilage MRI of other joints, such as ankle or hip, may also become feasible in the future. Generally, with whole-body ultra-high-field (B0 ≥ 7T) MRI systems finding more widespread use in research settings, advanced MRI methods such as gagCEST are also likely to gain relevance.

## 4. Selected Non-Destructive Quantitative Articular Cartilage Live-Imaging Methods

Due to the fact that current clinical non-invasive methods such as radiography and magnetic resonance imaging (MRI) are both somewhat limited in resolution and are not able to depict many of the molecular components of the articular cartilage ECM, there is a need to also focus on alternative imaging techniques and their clinical translation. One such method is based on the spectroscopy of the inelastic scattering of photons, known as Raman spectroscopy [124]. Using monochromatic light interacting with molecular vibrations, the releasing energy shift of the photons can be evaluated, and for cartilage tissue imaging, Raman spectroscopy can be improved to make use of non-linear optical effects by two or more wavelengths from synchronized pulsed lasers. Unal et al. [124] compared Raman spectroscopy-based water measurements in cadaveric human articular cartilage explants to gravimetric and MRI-based water measurements and developed a new method to assess the hydration status of cartilage non-destructively. The authors demonstrated an association of the Raman-based water and organic content measurement with the mechanical properties of the cartilage explant and concluded that Raman spectroscopy is able to provide water content information from native tissue without the need for dehydration and might be suitable for estimating mechanical cartilage function. A recent publication reviewed Raman spectroscopy techniques for ECM characterizations over a variety of exciting applications and tissue systems, including native tissue assessments (bone, cartilage, and cardiovascular), regenerative medicine quality assessments, and diagnostics of disease states [125].

Coherent anti-Stokes Raman scattering (CARS) and second harmonic generation (SHG) are additional non-linear techniques that allow label-free, non-destructive, and non-invasive imaging for cellular and tissue analysis [126]. The difference between CARS and usual Raman spectroscopy is that CARS utilizes multiple photons with molecular vibrations, resulting in a coherent and stronger signal, whereas SHG is unique in its ability to generate the second harmonic signal from non-centrosymmetric structures (e.g., from collagen, myosin, and microtubules) when excited by ultrashort femtosecond laser light. This provides highly specific molecular-level imaging by utilizing the endogenous signal to image collagen in cartilage at (sub-) micron-level resolution [126,127]. CARS imaging on the spectral region between 800 cm^−1^ and 1800 cm^−1^ represents the so-called ‘fingerprint’ region, which is rich in biochemical information on chemical functional groups related to tissue proteins, lipids, glycogen, and nucleic acids. The Raman band at 1061 cm^−1^ represents glycosaminoglycans with sulphate groups (OSO3^−^ symmetric stretch). The amide I band at 1668 cm^−1^ is mainly assigned to collagen, and the Raman CH vibrational mode at 1450 cm^−1^ (CH2/CH3) is related to collagens and proteins, which means that CARS signals from both fibrillar and non-fibrillar collagen types can be observed [126,128,129]. This is a significant difference from SHG, which is sensitive to micro-fibrillar collagen types. Boyanich et al. combined confocal, SHG imaging, and AFM microscopy to investigate the ultrastructure of the superficial zone [130]. The authors demonstrated that elastic fibers are most prevalent at the surface, where collagen and chondrocyte densities are lowest, and, at the interface of this most superficial layer with the underlying bulk cartilage, a dense fibrillar network exists, formed mainly by collagen fibrils and elastin microfibrils [130]. Studies such as this one illustrate that combining multiple imaging techniques allows taking advantage of the strong side of each of these technologies.

Moura et al. [126] monitored the chondrogenic differentiation of human fetal femur-derived skeletal cells in three-dimensional cultures using CARS and SHG microscopy and demonstrated the live imaging of the developing neo-tissues over time. The authors demonstrated that CARS microscopy does not alter the phenotype or the gene expression profile of the cells in their different stages of differentiation. Additionally, the imaging had no adverse effect on human skeletal cell growth and behavior due to cell damage or phototoxic effects when threshold levels were respected. This is an important aspect when considering using these imaging techniques in patients. Interestingly, CARS imaging at the 1668 cm^−1^ Raman band displayed a distinct collagen signal that was different when compared to SHG, with CARS being responsive to the molecular structure and chemical composition and SHG being sensitive to the super-molecular crystalline structure of collagen [126,131,132]. Subsequently, the authors concluded that multimodal imaging with non-linear techniques, such as CARS and SHG, offers new approaches for clinical translation for the assessment of regenerating skeletal tissues and is ready for implementation by biomedical scientists. Indeed, the first steps for the transfer of SHG imaging into an endoscopic setting have been made recently when real-time in vivo endomicroscopy images of the microsplits and ultra-structural fibril arrangements of collagen fibers were demonstrated in the articular cartilage of porcine knee joints [133,134]. However, a limitation of laser light-based imaging is a limited penetration depth, which might only allow the evaluation of more superficially located tissue areas [135,136].

In terms of diagnosing early degeneration of articular cartilage, SHG can detect the structural and morphological changes in collagen architecture and collagen types in early-stage cartilage damage by taking advantage of collagen specificity and high-resolution imaging [137]. Polarization-resolved second harmonic microscopy (p-SHG) goes even one step further by differentiating type I and II collagens, indicating that it can be utilized to differentiate articular cartilage from fibrocartilaginous repair tissue and, hence, demonstrates its capability for the assessment of cartilage repair monitoring [138].

Chondrocytes can be imaged by utilizing a two-photon excited autofluorescence microscopy (TPEF) signal, which can be optically separated from the SHG signal and further enhanced by using sodium fluorescein, an FDA-approved clinical dye, which allows the simultaneous assessment of collagen alteration, changes in cellular morphology, micro-cracks, and associated chondrocyte death patterns [138,139]. TPEF can also be used to probe the distribution of elastin fibers within articular cartilage [140]. Mansfield et al. also uncovered local patterns in organization of collagen fibers at the submicron scale in the context of the microstructural response of articular cartilage zones to mechanical loading and ECM disruption patterns close to chondrocytes in degenerated articular cartilage by using p-SHG [141,142]. However, the true pericellular matrix could not be resolved in articular cartilage under strain, and the underlying reason remains unclear. Whereas SHG imaging can be used for quantifying the intensity of micro-fibrillar collagens such as type I, II, III, V, and XI, it cannot be used for individually measuring these collagen types or type VI collagen. In this context, the authors of this review used SHG successfully to depict the micro-fibrillar collagen components of the human PCM in articular cartilage and uncovered OA-associated PCM damage in conjunction with proliferating OA chondrocytes that exit their PCM [67]. SHG imaging has recently been used to distinguish between live/dead chondrocytes by visual assessment [123], which could be vital to trauma-related investigations. Interestingly, the normalized autofluorescence ratio was proposed as a quantitative indicator to determine chondrocyte viability, which is possible because viable and dead chondrocytes exhibit signals with distinct normalized autofluorescence ratios [123]. However, it appears that a standard dye solution would be needed to account for different setups. Another study integrated stimulated Raman scattering (SRS), SHG, and two-photon excited fluorescence (TPEF) imaging into one system termed a “multimodal nonlinear optical (NLO) microscope system” [143]. In a subsequent study, the authors demonstrated in a mouse model that it was possible to visualize the fibrillar collagens within the ECM by the forward and backward SHG signals, while high-resolution imaging of chondrocytes was achieved by capturing endogenous TPEF and SRS signals of the cells [144]. Label-free imaging NLO microscopy is also applicable to live tissues.

Another interesting non-destructive approach is using hyperspectral microscopy, which is a novel technology that utilizes endogenous fluorophores to image tissue autofluorescence, followed by unmixing of the fluorescence signals of individual tissue compounds, to non-invasively assess the molecular composition of cells and tissues [145]. Recently, Mahbub et al. used this technology on healthy bovine articular cartilage and human OA articular cartilage in comparison with SHG imaging for collecting molecular data to characterize the state of OA and potential treatment effects [145]. The study demonstrated that the well-known differences in types I and II collagen between the superficial and transitional layers of healthy articular cartilage were visualized using this technology. The authors then simulated human OA cartilage treatment by adding an unfortunately proprietary composition containing secretions from adipose-derived human MSCs, with or without 6% *v*/*v* hyaluronan, and compared this to “untreated” controls. Hyperspectral microscopy was able to discriminate between the type I and II collagen contents of the control vs. treated OA cartilage. Whereas this is an impressive technological demonstration, the current limitation is that chemically pure reference fluorophores are required to define their spectral profile, and such substances are not yet commercially available for, e.g., type VI, VIII, and IX collagens. The reference method used in that study to confirm collagen autofluorescence was SHG, which is another interesting non-destructive live-imaging method, as discussed above.

Optical coherence tomography (OCT), which is a non-invasive and label-free imaging technique based on the backscattering of light, delivers images in real time and uses light in the near-infrared spectral range, with a tissue penetration depth of several hundred micrometers. We refer the interested reader to a detailed review [146] on principles and technical realization, which presents the topic in the context of retinal OCT. Polarization-sensitive optical coherence tomography (PS-OCT) is a functional PCT extension that is based on the polarization properties of light and quantifies tissue birefringence sensitive to the orientation and organization of collagen fibers [147,148]. Specifically, a reduced birefringence is associated with articular cartilage degeneration [149,150,151]. In this context, a recent study proposed an OA-evaluating method based on PS-OCT imaging, in which two parameters, namely phase homogeneity index (PHI) and zonal distinguishability (Dz), are used to quantify the signal fluctuation within each zone and the zone-to-zone variation of the tissue birefringence properties [148]. These parameters were then combined to generate a so-called GPS score, which correlated significantly with different stages of OA [148] that were determined histologically using the OARSI scoring system [8]. The authors concluded that the suggested method performs well in detecting tissue inhomogeneity and zonal distinguishability and has potential to be developed as a clinical tool for detecting OA [148]. Although articular cartilage OCT can depict structural morphology due to changes in tissue scattering properties [149,152,153], in contrast to SHG, it might be relatively difficult for OCT to visualize morphological changes associated with collagen fibers in early degeneration or post-traumatic damage due to a lack of collagen specificity. The differentiation between types I and II collagen can be performed more accurately in p-SHM than in polarized-OCT [127,138].

In another recent study, a polarized reflectance imaging setup as described in [154] was used to generate polarized reflectance maps to study how surface roughness, zonal collagen microstructure, and chondrocyte organization contribute to polarized reflectance signals in bovine articular cartilage [155]. Interestingly, the results indicated that polarized reflectance textures were derived from the superficial zone collagen network, while average values also depended on surface roughness and cartilage thickness. In contrast, the high correlation of polarized reflectance texture with split lines did not extend to a clear relationship with the organization of chondrocytes in the superficial zone, suggesting that polarized reflectance depends more upon collagen microstructure than scattering from cells [155]. Thus, polarized reflectance imaging appears to be valuable for collagen network imaging and surface roughness. In comparison with other clinical or potentially clinically applicable cartilage imaging methods, non-microscopic technologies such as CT and MRI have lower resolution, whereas PS-OCT and polarized reflectance imaging might compete with each other.

Whereas the above-discussed methods are mainly sensitive to collagen content and/or structure, a few other non-destructive methods have been established that are sensitive to both the collagen and proteoglycan contents of articular cartilage. An interesting method for imaging cartilage is using fluorescence lifetime readouts of cartilage autofluorescence, which was first reported in 2004 [156]. Technically, autofluorescence lifetime (AFL) measurements use a fluorometer that can be combined with an optical fiber probe extension that quantifies autofluorescence at 460 nm. Using a single-point time-resolved spectrofluorometer, a recent study demonstrated that AFL can provide a label-free optical readout of cartilage degradation because changes in AFL of ex vivo articular cartilage were detected in control cartilage vs. collagenase-, human MMP-1-, and trypsin-treated cartilage, and also in control cartilage vs. retinoic acid-treated cartilage for inducing production of aggrecanase, with the latter using porcine articular cartilage and bovine nasal cartilage [157]. A subsequent study with the view to developing a clinical instrument for the non-invasive monitoring of cartilage status investigated AFL sensitivity to chemically induced aggrecan depletion [158]. That study demonstrated that aggrecanase treatment of mouse and human cartilages reduced AFL and, therefore, can be used to detect areas of cartilage erosion [158]. AFL holds clinical potential because it can potentially be used in combination with arthroscopic devices. Furthermore, this approach might also be helpful in assessing cartilage mechanical properties because one study demonstrated a correlation between cartilage fluorescence intensity of the 330 nm excitation band and mechanical stiffness that was measured with a cylindrical 1 mm diameter indenter [159]. In another method, conventional Fourier-transform infrared microspectroscopy (FTIRM) of healthy bovine cartilage was also capable of detecting signals that arose from collagen and proteoglycans. For example, FTIRM has been used to identify the specific molecular components that contribute to its IR spectrum and, across the superficial, middle, and deep zones, the intensities of the absorbance bands that arise from the primary nonaqueous components of cartilage—such as collagen and proteoglycan (primarily aggrecan)—reflected the differences in the quantity of these specific components [160].

In summary, the methods presented in this text section highlight that a range of microscopic techniques are capable of delivering quantitative image information and are on the verge of translation into clinical use. The described techniques are either sensitive to the collagen network (SHG, PS-OCT, and polarized reflectance imaging) or to both the collagen and proteoglycan contents (AFL and FTIRM). In the future, it will become important to elucidate which of these techniques are sensitive to changes in the content of ECM components vs. structural changes such as collagen network damage without major content changes. It will also be important to test the resulting quantitative information against a well-accepted high-resolution grading system, such as the OARSI scale, in order to answer whether any of the promising techniques is or will be capable of statistically differentiating between OARSI grades 0 and 1, or at least between grades 1 and 2. Such capabilities would enable detecting of early degeneration. Finally, with the goal in mind to develop articular cartilage imaging into non-invasive quantitative functional information, this and the previous text sections together demonstrate that multiple microscopic techniques have been developed that can deliver quantitative imaging data. Whereas functional cartilage imaging appears to be centered on MRI technology for investigating the mechanical loading of articular cartilage, microscopic technologies for generating functional data are still lagging behind. Lastly, as discussed above, not all technologies investigate the same structural or functional targets. Thus, which one of the multiple technologies will be ‘the winner of the race’ to one day clinically detect early pre-clinical cartilage degeneration with quantitative functional imaging data remains unknown. As of today, this question cannot be answered because (i) we still do not understand the initiator(s) behind the mechanisms of early cartilage degeneration and (ii) OA encompasses a range of pathomechanisms and, thus, is a multifaceted and heterogeneous syndrome [161] with different phenotypes of structural damage [162].

## 5. The Articular Cartilage Image as Non-Invasive Functional Information for AI Classification of Degeneration and Prediction of OA Progression

Studies are increasingly using artificial intelligence (AI) methods for the image-based detection of early cartilage degeneration, early OA diagnosis, and OA progression, in part recently reviewed in [163], which illustrates the increasing importance of AI in this field. In the context of cartilage degeneration, studies used AI for predicting the OARSI classification of cartilage explants from MRI signals [105,164]. For example, OARSI score prediction with clinical MRI scan types that are sensitive to cartilage matrix changes yielded accuracies of up to 86% and R^2^ values between 0.65 and 0.85 with multiple linear least-squares regression, whereas binary classification was less successful [164]. Another study identified that the best classifiers for OARSI grade and score were T1- and T2-weighted image intensities and reported accuracies of 68% and 75% [105]. Together, these studies demonstrated that AI could discriminate between MRI scans of normal vs. pathological human articular cartilage explants. In a clinical context pertaining to OA, the main type of data that was used in conjunction with AI was imaging; 61% of articles used X-ray and 22% used MRI data [163]. One study analyzed pairs of weight-bearing knee X-rays by comparing initial X-rays that were scored as normal (Kellgren and Lawrence (K&L) classification system: grade 0) and X-rays that were taken approximately 20 years later [165]. The study demonstrated 62% accuracy in predicting from initial X-rays whether OA progression would result in a K&L grade of 2 and 72% accuracy in predicting a K&L grade of 3. A few MRI studies used image-derived data as AI input by investigating cartilage texture features [164,166,167,168]. Interestingly, one study used isolated T_2_ maps for classifying which individuals had or had not progressed after three years to symptomatic OA with 75% accuracy [168], whereas another study used cartilage texture maps and classified the progression to symptomatic OA with 78% accuracy [166]. Another study investigated MRI- and X-ray-based features of knee OA structural progressors and identified the best features in conjunction with the sparse partial least square method [169]. Collectively, these studies clearly demonstrated that cartilage images are suitable data for AI classification and prediction.

## 6. Introducing a Future-Oriented, AI-Supported, Non-Destructive Quantitative Optical Biopsy for Early Disease Detection

Our contribution to the field is the introduction of a future-oriented, AI-supported, non-destructive quantitative optical biopsy [4] that recognizes specific stages of the superficial chondrocyte spatial organization (SCSO) and, thus, operates on a digital tissue architectural fingerprint. By way of explanation, the SCSO can be conceptualized as follows: while standing on a forest floor or while viewing forest trees when standing on a ladder, you would not see much except for some tree trunks. Moreover, you would not be able to recognize any patterns in the organization of trees. If you sat in a helicopter and you looked down onto the forest, you would probably also see nothing except green treetops. However, if you replaced the trees with a map of tree positions, you would start recognizing patterns of spatial organization, such as trees that aggregate in valleys around water but not on high, bare hilltops. In geology, it is a common approach to consider spatial data. Comparably, we use the top view onto the articular cartilage surface to visualize cell arrangements and, in a reductionist approach, to visualize the chondrocytes but not the ECM between the cells. Using these images, we categorized the visualized SCSO types according to our initial work [25,53] and then analyzed the mathematical positions of cells in a 2D or 3D coordinate system and used this information as a quantitative fingerprint of digitalized tissue architecture. Thus, we termed this fingerprint the ‘SCSO’, as it captures the superficial chondrocytes’ spatial organization.

While this is interesting, the potential impact of this approach only comes to mind if one considers the briefly summarized major characteristics described here that are associated with the SCSO. Throughout our work, we elucidated that (i) the SCSO displays a high level of complexity, is species-, joint type-, and surface-specific articular cartilage [25], and thus can be used to discriminate between cartilages from different anatomical joint locations, purely by comparing joint surface images. Moreover, (ii) the SCSO is not inborn or stable but, instead, changes dynamically during the lifetime of an individual, as our lifetime model of the SCSO revealed [67], illustrating that the SCSO is responsive to its environment. Importantly, (iii) the exact characteristics of the SCSO depend on whether a given articular cartilage sample has been obtained from a healthy joint, whether it is situated in the macroscopically intact cartilage neighborhood of an early pre-clinical focal lesion, or whether the sample has been derived from within an OA lesion of an OA patient joint [53,62]. Because the SCSO undergoes distinct remodeling processes in response to early focal OA, even in the remaining, intact cartilage of a joint [53,62], the SCSO can be used to diagnose early OA onset before OA progression results in tissue damage and, thus, likely before clinical symptoms [62]. Providing longitudinal data on SCSO dynamics over time, we used FGF-2 in a prior study for inducing chondrocyte proliferation within early OA cartilage explants [67], which not only induced a structural phenotype similar to advanced OA but, more importantly, recapitulated the full range of loss of SCSO that can be observed in human OA. Based on such disease stage-typical SCSOs, the SCSO can be used to (iv) discriminate cartilage explants. In fact, our laboratory uses such SCSO characterization of live cartilage samples prior to other measurements, e.g., when using hybrid fluorescence-atomic force microscopy to scan cartilage degeneration across length scales [57] or for stratification and generation of experimental groups, e.g., when assessing surface nanoscale stiffness of the joint surface as a function of the SCSO [4]. However, perhaps the most important point is that (v) the SCSO can be used to calculate quantitative SCSO stage-specific spatial data [54,67,68]. Based on the quantitative nature of such data, this approach allows correlating the SCSO with other types of quantitative information, such as molecular, cellular, and clinical data, including patient scores. This opens the door for studies that assess diseases such as OA on multiple scales, e.g., from the molecular level to the patient level, which would be helpful for better understanding early disease states, as many basic science studies are naturally disconnected from clinical data, are not tissue-, joint-, or patient-specific, and, in short, are contextually non-specific.

**Figure 3:**
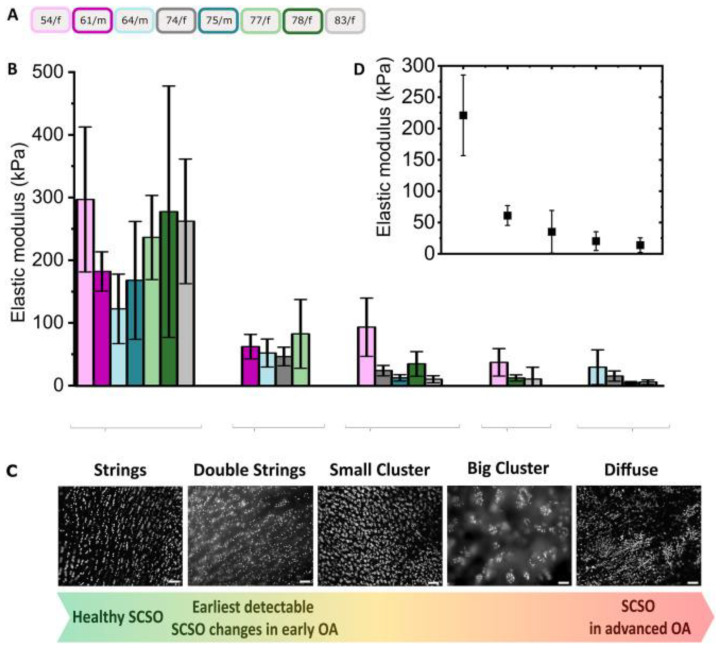
Correlation of fluorescence and AFM-based data. (**A**) Color-coded patient age and gender. (**B**) The elastic moduli of eight OA patients. Each bar represents the mean elastic modulus of the articular surface of an AC disc (3 regions of interest (ROI) per disc, 256 force vs. indentation curves per ROI). The respective error bars indicate the standard deviation. (**C**) The corresponding predominant SCSO of each AC disc. Given are fluorescence images of DAPI-stained nuclei of superficial-zone chondrocytes displaying distinct stages of healthy (strings) and OA-related SCSOs. Scale bars: 100 μm. (**D**) The patient-averaged elastic modulus. AFM indentation data showed a strong correlation with the SCSO (*r_rm_* = −0.91, 95%CI: −0.97, −0.73, *p* < 0.000002 for linearly transformed disc averaged data elastic modulus vs. SCSO data). The elastic moduli differed between the string SCSO and all other SCSO stages (*p* ≤ 0.001). This figure is a reprint of Figure 2 published in [4]. Permission for this reprint has been obtained.

However, in order to realize such goals, it became necessary for us to generate statistically usable quantitative SCSO data with clinical technology. In this context, our team has recently demonstrated that medically approved technology can be successfully used for visualizing and quantifying the SCSO [4]. Utilizing this approach, we recently delivered the proof-of-concept that early OA pathology detection is indeed possible with the currently available clinical technology [4]. In brief, our strategy was to focus on articular cartilage surface stiffness because loss of stiffness is an early OA pathology that occurs well before morphological changes [170] and is a sensitive indicator of the initiation of PTOA [171]. Thus, we measured nanoscale stiffness changes as a function of the SCSO and established the SCSO as a visualizable surrogate marker of loss of articular cartilage surface stiffness [4] (Figure 3).

With the goal of introducing SCSO data as the quantitative backbone of a model-based optical biopsy for detecting early OA, we defined specific SCSO types as spatial point patterns, as described previously [54,67,68], and calculated spatial data that we used as training input for a machine-learning random forest (RF) model. In detail, the chosen RF model correctly determined in 100% of samples the SCSO of both the test subset and the samples that were recorded with clinical technology. In turn, this allowed determining for a particular cartilage sample its associated loss of surface stiffness, which was used here as an example of early OA pathology that is otherwise difficult to assess clinically and non-destructively. Thus, in short, we recently introduced a future-oriented, AI-supported, non-destructive quantitative optical biopsy for early disease detection [4]. In the context of this review, this approach represents a step towards altering the usage of articular cartilage structural images towards an image-based biomarker that is associated with quantitative functional information. Moreover, because clinical trials for OA are still unable to recognize the early, potentially therapeutically addressable disease stage, and because disease-modifying drug candidates can subsequently not yet be tested this early in the disease, our recently introduced AI-supported, non-destructive quantitative optical biopsy might be helpful in contributing to solving a major problem in our field.

## 7. Conclusions

Molecular advances in orthopedic trauma diagnosis and therapy are relevant for transformative improvements in patient outcomes. However, such advances are highly dependent on our capability of visualizing the structural and functional changes in cells and tissues in the early stages of disease(s). This review discussed the structural targets that are relevant in articular cartilage imaging and presented how articular cartilage imaging has undergone profound changes based on significant improvements of the existing imaging technology, e.g., MRI, and summarized novel techniques, e.g., advanced live imaging. Together, these exciting changes have led, over time, to a noticeable change in the purpose and expectations for which cartilage imaging is being conducted. Basic science offers multiple designated and highly advanced imaging methods for assessing the early stages of disease, whereas clinical imaging is lagging behind, focusing on early symptoms. To aid in closing the gap between the two basic science and clinical ‘imaging fields’, we have explored how advances in technology, strategy, and artificial intelligence (AI) are increasingly shaping the way articular cartilage is imaged. We conclude that the structural cartilage image is progressively being transformed into functional data. This capability, in conjunction with AI classification and prediction, would aid translational diagnostic applications and the development of preventive or early therapeutic interventions that are yet beyond our reach.

## Figures and Tables

**Table 1:** Structural targets for articular cartilage imaging.

Targets	Details	Text Sections
Extracellular matrix (ECM)	Collagens (types, zonal organization, disruption) Ground substance (proteoglycans and GAGs, content and zonal distribution, water content)	Text Section 2.2
Non-mineralized zones of articular cartilage	Superficial (surface integrity, fibrillation, fissures, cracks) Transitional/middle Radial/deep	Text Section 2.3 and Section 2.4
Cartilage thickness	Variation (joint-dependent, age, sex) Reduction (lesions, tissue loss)	Text Section 2.5
Articular chondrocytes	Morphology (volume, shape) Superficial chondrocyte spatial organization (SCSO) (strings, double strings, clusters, diffuse) Zonal differences Vitality (types of cell death: necrosis, necroptosis, apoptosis, hypo- or hypercellularity) Hypertrophy	Text Section 2.6, Section 2.7 and Section 2.9
Pericellular matrix (PCM), Chondron	Morphology (shape, integrity, composition)	Text Section 2.8
Hypertrophic zone, tidemark, cement line	Doubling, integrity (high-density mineral infills (HDMI); cracks and high-density mineralized protrusions (HDMP)	Text Section 2.9
Subchondral bone (SB)	Bone plate (microfractures, edema, vessel ingrowth) Trabecula	Text Section 2.10

## Data Availability

Not applicable.
